# Explaining the Slow Adoption of AI Innovations in Health Care: Network Analysis Approach

**DOI:** 10.2196/60458

**Published:** 2026-02-23

**Authors:** Petra Apell, Sara Locher, Annie Milde, Henrik Eriksson

**Affiliations:** 1 Department of Technology Management and Economics Chalmers University of Technology Göteborg Sweden; 2 Department of Engineering Science University West Trollhattan Sweden

**Keywords:** artificial intelligence, health care, medical device, technological innovation systems, TIS

## Abstract

**Background:**

Artificial intelligence (AI) is a topic of considerable hype, with many actors sensing its high potential for health care applications. Despite this, the adoption has been slow, with few applications being implemented in clinical practice.

**Objective:**

The aim of our study was to investigate the challenges associated with using AI in health care, as well as provide suggestions for how further adoption of AI within health care organizations can be facilitated.

**Methods:**

A qualitative case study with a mixed methods approach was conducted at one of Sweden’s largest hospitals. Regulatory approved AI medical devices were analyzed, and primary qualitative data from 14 expert interviews were collected and cross-referenced with secondary quantitative data. The framework of technological innovation systems was used to analyze the system factors and their dynamics to identify blocking mechanisms and areas for improvement.

**Results:**

The challenges related to knowledge development, diffusion, legitimation, and resource mobilization could trigger a cascade of positive activities, thereby significantly enhancing the overall performance of the innovation system. Creating dedicated testing environments to evaluate safety and efficacy would facilitate the routine clinical use and reinforce the use of AI innovations in health care organizations.

**Conclusions:**

This analysis shows that the adoption of AI health care technology innovations can be accelerated through targeted strategies and supportive mechanisms triggering virtuous cycles that facilitate clinical validation and generate compelling use cases. The interconnection between guidance of search and entrepreneurial experimentation has been confirmed, providing the initial conditions for knowledge development, diffusion, and legitimation in the early stages of emerging technologies.

## Introduction

### Background

Emergent technologies in health care, such as artificial intelligence (AI), can transform health care [1], and AI has been recognized as a key source of economic growth and societal development by the Swedish Agency for Digital Government [2], particularly for the public sector, with a potential annual savings of up to €12.5 billion (US $14.6 billion) equivalent to 6 percent of total public spending. Despite technological advancements, digitalization within the health care sector lags behind that of other industries, and the adoption of AI technologies has been slow [3]. While the industry is considered a primary driver for the digitalization of early-stage digital health startups [4] and well-established life science companies [5], most AI initiatives in the Swedish health care system were related to research projects [6]. Capturing the full value of AI technology involves numerous obstacles and challenges, such as ethical considerations [7,8] and regulations [9,10], and lessons learned from implementation and innovation sciences suggest that each individual factor is less important than the dynamic interactions between different factors [11,12]. The greater the complexity of an innovation or its context, the lower the likelihood of successful adoption, scaling, dissemination, and long-term sustainability [13,14]. The primary purpose of this study is to explore the opportunities and challenges associated with the integration of AI in a health care context, while also providing recommendations to facilitate its further adoption.

### Research Questions

To unravel the complexities surrounding the adoption of AI technologies, we have used the Technological Innovation System (TIS) framework [15-17]. This conceptualization views technological systems as intricate networks of agents operating within an economic or industrial domain, influenced by a specific institutional infrastructure. Applying the TIS framework to evaluate the limited adoption of AI innovations within a specific health care organization helps to evaluate the dynamic interplay of actors and institutions in the AI adoption landscape within health care systems. The following research questions were formulated:

1. RQ1: What are the primary strengths and challenges associated with the adoption of AI innovations in health care?
2. RQ2: What are the patterns and interrelationships among the key factors and processes that influence the adoption of AI technology innovations?
3. RQ3: What system-blocking mechanisms are related to the adoption of AI technology innovations in health care?

### AI Technologies in Health Care

While AI holds promise in various health care applications, its current prominence is particularly evident in imaging analysis within the field of radiology, facilitating the analysis of large image datasets [18,19]. Historically, health care has been designed to treat entire populations with the aim of developing solutions that can address the needs of large groups of individuals with similar symptoms; however, a current trend suggests that AI is expected to soon drive a significant shift toward precision medicine (also referred to as personalized medicine) [20]. Future developments are expected to include advanced telemedicine, including predictive and preventive self-diagnostic medical devices [21]. Mobile AI devices can provide personalized support to maintain healthy behavior, valuable insights to health care providers, and increased adherence to prevention programs [22].

### TIS Framework

The TIS framework is a leading approach for analyzing emerging technologies, focusing on the structures and functions that shape innovation activities [15-17]. The structural TIS components incorporate actors, networks, and institutions, encompassing both formal elements (such as laws) and informal elements (such as norms) that collectively shape the “rules of the game” [16]. Table 1 outlines the key system functions, each of which influences the overall performance of the TIS. The strength of these functions can be evaluated using indicators, such as (1) changes in available resources for the technology, (2) qualitative analysis of end user needs, and (3) number of scientific publications, or (4) establishment of international standards [15-18,23-25]. The system function of knowledge development and diffusion (F1) pertains to the depth and breadth of scientific, technological, and market knowledge and can be assessed by analyzing the number of relevant publications [15,16]. The system function legitimation (F2) relates to the establishment of institutions that support a particular technology and the overall social acceptance of that technology. Indicators of function strength include the time from development to customer installations and qualitative data on the influence of legitimacy [15,16]. In this study, the function strength was assessed based on the perception of legitimacy and how it influences demands and behaviors. Resource mobilization (F3) assesses the ability to mobilize infrastructure, financial capital, and human resources. Changes in resource volumes related to the innovation system are indicators of function strength [15]. The system function guidance of search (F4) guides innovators and relates to incentives and expectations of growth potential. The function strength can be assessed by qualitative analysis of end user needs of the particular technology [15]. Entrepreneurial experimentation (F5) involves probing new technologies and applications, while function strength can be assessed by the number of applications, new entrants, and actors [15]. The function market formation (F6) concerns the intersection of supply and demand, and its strength can be assessed by analyzing the stage of market maturity and the factors that drive market development, such as the existence of public tenders and reimbursement [26]. The function system-wide synergies (F7) captures the conditions necessary for fostering collaboration across the innovation system. Its strength can be assessed by examining the establishment of international standards and the formation of formal networks among actors, both of which contribute to improved technology interoperability and broader acceptance [15,16].

**Table 1:** Innovation systems, functions, and strength indicators.

System function	Function description	Indicators of function strength
F1: Knowledge diffusion and development	Breadth and depth of scientific, technological, and market knowledge base	Market and technological knowledge assessed by experts. Scientific knowledge is assessed by the temporal development of the number of publications in scientific journals.
F2: Legitimation	Social acceptance and compliance of the technology with relevant normative institutions	Perception of legitimacy and social acceptance for the technology. Temporal development of the number of articles in daily newspapers.
F3: Resource mobilization	Mobilization of infrastructure. Human and financial resources.	Availability of capital and the perceptions of whether resources are sufficient.
F4: Guidance of search	Degree of influence on the development direction within the innovation system	Belief in growth potential and articulation of demands and needs for the technology by end users.
F5: Entrepreneurial experimentation	Probing and testing of new technologies and applications	Number of new entrants and actors. Availability of AI^a^ technologies in the medical fields and the diversity of applications.
F6: Market formation	Refers to the market of available products and the availability of innovations	Size/ type of markets/customers, and actors’ strategies to enhance market access.
F7: System-wide synergies	The extent to which system functions reinforce one another can be viewed as an indicator of the overall dynamics of the system	Establishing standards and formal networks between actors.

^a^AI: artificial intelligence.

## Methods

### Literature Review

An iterative search was conducted in Scopus, Web of Science, and Google Scholar, supplemented by gray literature, such as government reports and white papers from organizations, including the World Health Organization (WHO), Organisation for Economic Co-operation and Development (OECD), the European Commission, and national health ministries. Sources were selected for their conceptual relevance to the TIS framework and applicability to health care. A combined narrative and integrative review approach enabled the flexible inclusion of diverse materials, and the insights informed the case study design and subsequent empirical analysis.

### Research Design and Analytical Approach

A case study was conducted to examine the adoption of AI technologies in health care using the TIS framework [27]. The aim was to analyze key system functions, processes, and dynamics associated with the development and diffusion of AI in the health care sector. We applied an abductive approach [28], operationalized through systematic combining [29], which enabled iterative movement between theory and empirical data throughout the case study.

### Research Setting

The case study was conducted at Sahlgrenska University Hospital, one of Sweden's largest university hospitals, which engages in advanced research across various medical disciplines. The Swedish Government (2018) has emphasized the importance of harnessing the potential of AI, aiming for Sweden to lead in technological development [26]. The decision to focus on Sahlgrenska was motivated by both data accessibility and Sweden’s consistently high ranking on the Global Innovation Index [30].

While the study focuses on Sweden, its ambition is to provide insights relevant beyond the national context. Because the dynamics of a specific innovation system are often linked to broader sectoral structures, the TIS is situated within a wider European context [31]. Accordingly, European data were incorporated, reflecting the interwoven institutional frameworks between Sweden and the European Union (EU), particularly in regulatory approval of AI technologies. The analysis focuses on structural components of the innovation system—actors, networks, and institutions—related to AI technologies in health care, specifically those applied within patient-facing departments, excluding administrative applications.

### Data Collection

Data collection was carried out in two phases: expert interviews and secondary data.

#### Phase 1 –Expert Interviews

Exploratory interviews with key stakeholders were first conducted to refine the project scope, increase contextual understanding, and familiarize the research team with the organization. This was followed by semistructured interviews, guided by a predefined set of questions, to balance direction and flexibility and capture diverse viewpoints [32]. Experts from multiple hierarchical levels were included to achieve triangulation and capture a comprehensive picture of the field. Purposive and snowball sampling were applied to identify relevant experts. A total of 14 expert interviews were conducted via Zoom (Zoom Communications, Inc) and Teams (Microsoft). One author led the interviews while another took notes, and all interviews were recorded to ensure complete and accurate data for subsequent analysis.

#### Phase 2 –Secondary Data

Secondary data were collected online and analyzed quantitatively, as summarized in Table 2. These data were used to cross-reference the primary qualitative findings and served as evaluative benchmarks for assessing functional strength. For example:

1. Knowledge development and diffusion (F1): assessed through bibliometric analysis of scientific publications related to AI in health care in both Swedish and European contexts.
2. Legitimation (F2): examined through the number of articles published in Swedish daily newspapers as an indicator of societal acceptance.
3. Guidance of search (F4): assessed through the temporal development of editorials on AI innovations in healthcare in European and Swedish journals.
4. Entrepreneurial experimentation (F5) and market formation (F6): analyzed through product mapping of medical devices under EU regulation (EU 2017/745) [33]. Products were categorized by purpose (Table 3), with some requiring subjective assessment due to overlap (eg, telemedicine and monitoring). Classification as AI was based on company descriptions in the absence of a standardized definition.

**Table 2:** Quantitative data for cross-referencing qualitative findings and assessing function strength.

System function	Quantitative data
F1: Knowledge diffusion and development	Scientific and technological knowledge was assessed by analyzing the temporal development of the number of publications related to the research topic in Swedish as well as European scientific journals.
F2: Legitimation	The temporal development of the number of articles related to AI^a^ innovations in health care in daily Swedish newspapers.
F3: Resource mobilization	Not quantitatively assessed in this study.
F4: Guidance of search	Temporal development of the number of editorials related to AI innovations in health care published in European and Swedish scientific journals.
F5: Entrepreneurial experimentation	A product mapping of approved medical devices was used to analyze the diversity of end user applications (supply side), and the number and variety of AI experiments for different medical disciplines were used to assess the level of engagement for the demand side.
F6: Market formation	A product mapping of medical devices was performed to analyze the number of available health care–related AI products in different categories based on their purpose. The number of commercial actors was also analyzed.
F7: System-wide synergies	The availability of established standards and formal networks between actors.

^a^AI: artificial intelligence.

**Table 3:** Product purpose categorization.

Product purpose	Description
Decision support	Advanced analysis
Diagnosis	Tools to detect correlations between symptoms and diseases
Monitoring	Aids for the monitoring and assessment of conditions
Telemedicine	Applications for remote health care
Workflow	Enhancement of workflow, such as triage and automation of tasks
Other	Robotics, prosthesis, etc

### Data Analysis

All expert interviews were transcribed prior to analysis. Transcripts and field notes were coded and analyzed in NVivo (QSR International) software to support the systematic organization of the data. Initial coding of first-order concepts was followed by aggregation into broader second-order themes and subsequently into overarching dimensions. As theoretical insights emerged, transcripts and field notes were revisited in iterative coding rounds to ensure rigor and transparency in the qualitative analysis [28].

The resulting themes and concepts were then examined using the TIS framework, drawing specifically on predefined indicators of functional strength (Tables 1 and 2). This study builds on earlier research on AI in health care using the TIS framework [5,24,25], as well as on empirical data and analyses reported in a master’s thesis by 2 of the authors [34].

### Ethical Considerations

This study involved the analysis of publicly available gray literature, including governmental reports and policy documents, and a limited number of exploratory expert interviews with stakeholders in their professional roles. According to the Swedish Act concerning the Ethical Review of Research Involving Humans (SFS [Svensk författningssamling] 2003:460), ethical review is required only when research involves sensitive personal data, physical interventions, or affects participants’ physical or mental integrity. As this study did not involve any such elements, formal ethical approval was not required. All participants were informed about the study’s aims, their participation was voluntary, and verbal informed consent was obtained prior to each interview. All interviewees were anonymized to protect their privacy and integrity. Recordings were made only with participants’ explicit consent to ensure accurate interpretation. The research was conducted in accordance with good research practice as outlined by the Swedish Research Council.

## Results

### Actors, Networks, and Institutions

**Figure 1:**
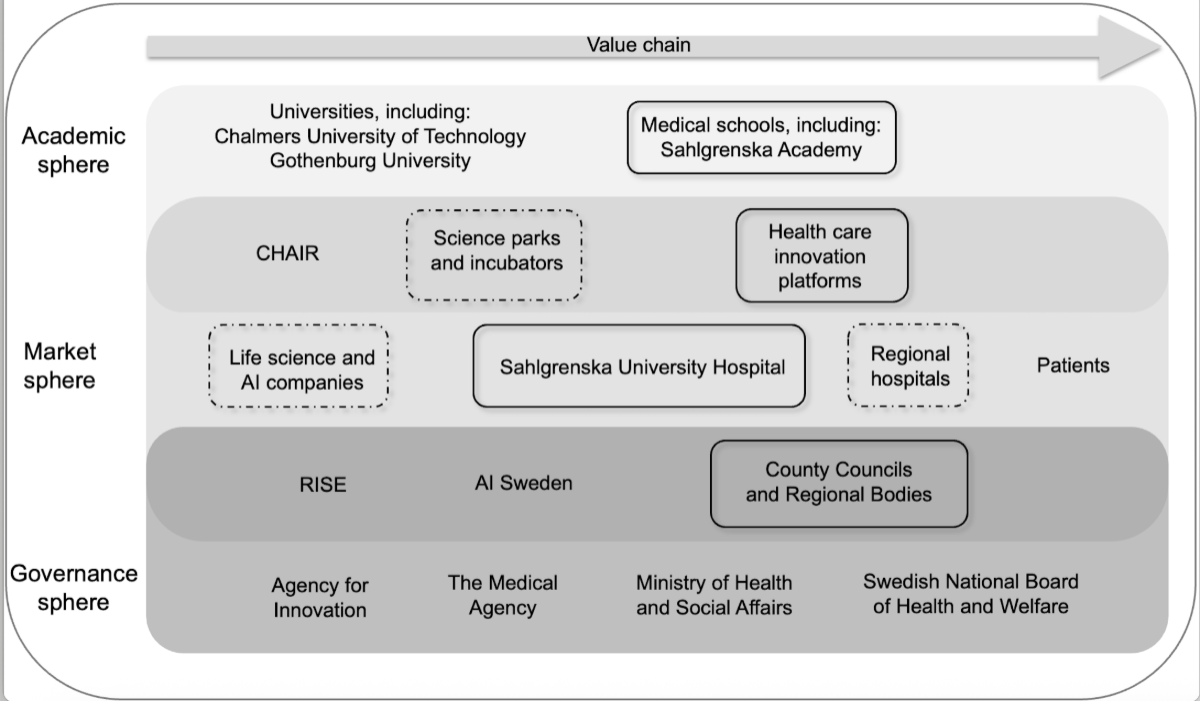
Selection of central actors, networks, and institutions related to artificial intelligence technologies in a health care context.

An analysis of the structural components influencing the regional innovation system is illustrated in a regional innovation system map categorized into academic, market, and governance spheres along the value chain, as shown in Figure 1. Actors represented by solid-line boxes participated in both the exploratory meetings and the semistructured interviews. Those outlined with dotted lines took part only in the exploratory meetings to gain a deeper understanding of the research area.

Sahlgrenska University Hospital is a key actor within the market sphere, alongside life science firms, AI technology companies, and regional hospitals. The academic sphere included the central actors, Gothenburg University and Chalmers University of Technology, with its research center for AI (Chalmers Artificial Intelligence Research Centre; CHAIR). Innovation platforms, science parks, and incubators have been established to foster innovation and to actively facilitate the development of novel ideas and technologies. Research Institutes of Sweden (RISE) is an independent, state-owned research institute that serves as an innovation partner for academia, and private and public sectors. Among the national actors in the governance sphere were the Ministry of Health and Social Affairs, the Medical Agency, the Agency for Innovation Systems (Vinnova), and the Swedish National Board of Health and Welfare. The AI Sweden ecosystem network, which is the Swedish National Center for Applied AI, is funded by the Swedish government, as well as partners in the Swedish public and private sectors and aims to accelerate AI usage [35].

The regional structure has not benefited this area; quite the opposite. There is considerable competition among the regions. Everyone wants to be in the lead. There is amazingly little cooperation between the regions today.

Study participant A

### Functional Assessment

The section describes the key strengths (S) and weaknesses (W) associated with the adoption of AI innovations in health care.

#### Knowledge Development and Diffusion (F1)

Rather than gaining deep technological knowledge, many respondents emphasized the importance of in-house competence to test and validate products that could potentially be deployed in clinical practice. A recurring weakness theme was the lack of knowledge and expertise concerning the potential impact of AI at both the organizational level and in everyday clinical practice (W1)*.* Individuals at various levels within the organization were perceived to lack an understanding of the technological capabilities, which hindered their ability to articulate their needs and to use and implement AI in clinical practice. Furthermore, respondents emphasized the importance of enhanced competence among decision-makers and regional purchase units to increase the understanding of the market and commercialized products in order to bridge the knowledge gap and ensure sufficient expertise between internal and external actors.

While we need our own technical competence, we won’t spend time writing algorithms. This should rather be done by those at the Technology University.

Study participant C

In addition to collaborations with external actors, numerous research projects within the organization contributed to knowledge expansion (S1) and were considered a strength; for example, the Swedish Cardiopulmonary Bioimage Study, a massive and distinctive national initiative involving collaboration among 6 Swedish universities and university hospitals. The primary goal of the project is to predict and prevent chronic obstructive disease and cardiovascular disease by analyzing image data from 30,000 individuals. Moreover, the large number of research projects in other industrial sectors and the technological knowledge base among academia and the electromobility sector are broad. Knowledge diffusion from other sectors and among actors generated spillovers to the health care sector, but only to a limited number of individuals.

Machine learning is here to stay. Once you have opened that door, there is no going back.

Study participant K

**Figure 2:**
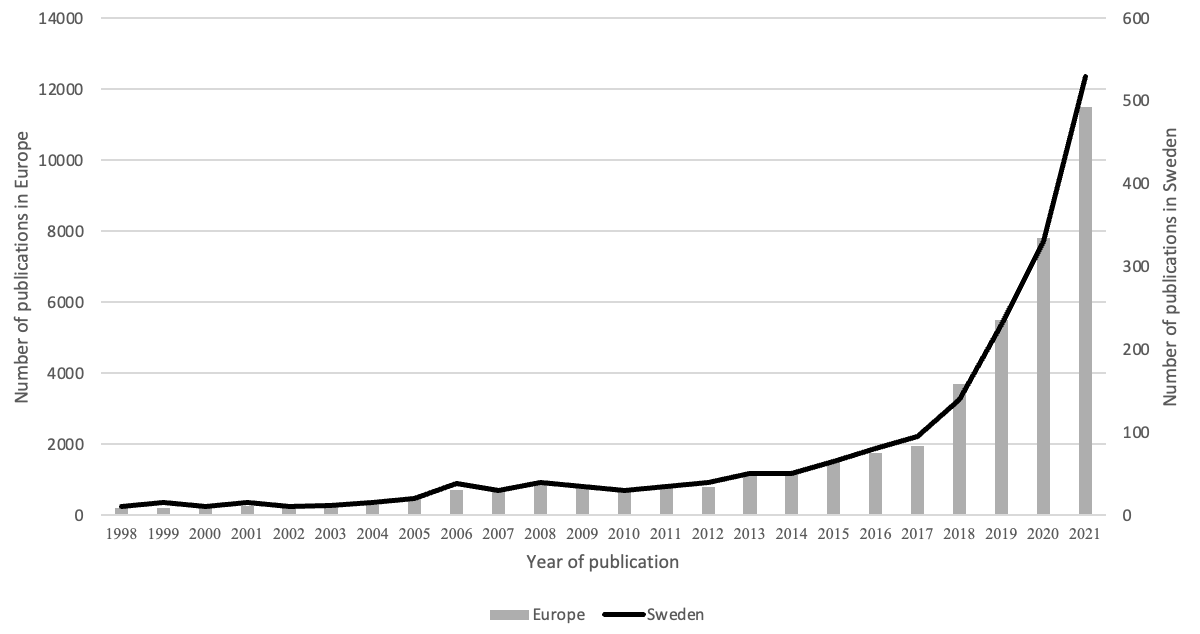
The number of published scientific articles addressing artificial intelligence in health care in a Swedish and European context (1998-2021).

To quantitatively assess the strength of the function, the number of relevant publications was analyzed. The number of publications in scientific journals has increased exponentially, which indicates a continuous expansion of the general knowledge base (S2); see Figure 2. Interestingly, the Swedish data exhibited a pattern similar to that of the European data, indicating that the scientific and technological knowledge developed along comparable trajectories.

#### Legitimation (F2)

In 2018, the Swedish Government unveiled its national strategy for AI, emphasizing the need to harness the advantages and calling for active support to develop and adopt the strategy (S3). RISE has developed an agenda that aims to expedite AI advancements in Sweden, with an emphasis on the role of AI in the public sector [36]. Moreover, international entities, such as the EU, assert the critical importance of adopting the technology, especially within health care [37].

It is a strategically important area for the hospital. We think it will be important to manage the challenges that health care is facing.

Study participant E

Hospital management, clinicians, and researchers all expressed a positive attitude toward the utilization and implementation of AI in health care (S4). Many interviewees highlighted the challenges of change management in health care, emphasizing that while individuals may hold a positive view of AI, they may resist the necessary activities and processes that are involved in reaching the end goal. The consensus-driven decision-making climate was viewed as potentially hindering transformative efforts, and some respondents called for more assertiveness and clarity in decision-making to drive necessary transformations (W2).

I think we have an anxious climate when it comes to decision-making. We are highly consensus-driven and that is not necessarily the best way to drive transformations. It is likely that some people will have to step forward and be clear about the fact that we have to do this, and then get people to follow.

Study participant D

One of the dominant themes identified in the interviews was the inherent complexity of the implementation of AI in health care, leading to uncertainties surrounding ethical considerations and patient data accessibility (W3). Participants expressed frustration over the difficulties of obtaining data due to the stringent compliance requirements, and data protection regulations, such as the General Data Protection Regulation (GDPR) and the Medical Device Regulation (MDR), were highlighted as major obstacles to the effective use of AI. Moreover, the weak transparency of data used for training the algorithms resulted in uncertainties regarding the reliability of AI innovations (W4). For example, issues with data quality, particularly concerning unstructured free text in medical journals, were emphasized, and the unease about algorithms trained on data from specific regions or populations and the ethical implications of such biases, questioning accountability when AI applications make mistakes, were emphasized. One participant shared an example of a diagnosis application that overlooked a rare condition due to limited training data, leading to concerns about responsibility in the event of errors.

**Figure 3:**
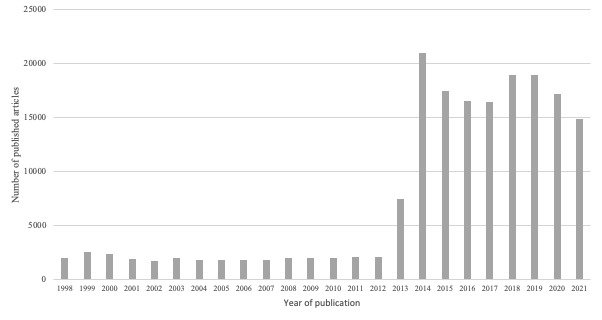
The number of published articles in Swedish daily newspapers (1998-2021) related to artificial intelligence innovations in a health care context.

To quantitatively assess the function strength, the temporal development of the number of articles in daily Swedish newspapers was analyzed. As Figure 3 illustrates, the number of articles increased exponentially, indicating an increased social acceptance of AI innovations in a health care context (S5).

#### Resource Mobilization (F3)

Respondents highlighted concerns related to access to patient data, the adequacy of digital infrastructure, and the interoperability of existing IT systems (W5). One interviewee pointed out the risk of compromising personal integrity, whereas individual patients can be identified with a sufficient number of anonymized data points. Additionally, the interviewees highlighted challenges related to data sharing, expressing frustration that health care data cannot be readily shared with external actors possessing high expertise in AI.

If AI is the rocket, data is the fuel.

Study participant K

A few participants identified financial constraints as a major challenge. In fact, some argued that obtaining funds was straightforward from internal and external organizations, like Vinnova (S6). However, individuals from hospital management acknowledged the difficulty of balancing strategic considerations with operational concerns, thus justifying that investments in areas with high uncertainties can be challenging. Furthermore, the recurring theme of health care staff facing considerable time pressures results in insufficient time to systematically explore potential AI applications (W6)*.* For example, despite being purchased and demonstrating added value, an AI-based software for magnetic resonance imaging had not been fully implemented in clinical practice due to time constraints*.*

The further down in the organization you are, the more occupied you are by daily problems.

Study participant A

#### Guidance of Search (F4)

Interviews revealed high expectations and a strong belief in the strategic potential of AI within the hospital (S8). There was a desire among the hospital management to be at the forefront of development and that AI should address real-world problems and provide value by improving health care and supporting the profession. For example, optimism was expressed about the potential to save time, automate tasks, enhance the overall quality of care, use telemedicine, and provide a “second opinion” in decision-making.

On one hand, you struggle with the strategic. On the other hand, you struggle with the operative. People in my type of position are expected to think of the strategy, but we are also occupied by operative issues, especially during a pandemic, when we must focus on solving acute problems. In such circumstances, these types of questions become overshadowed.

Study participant C

**Figure 4:**
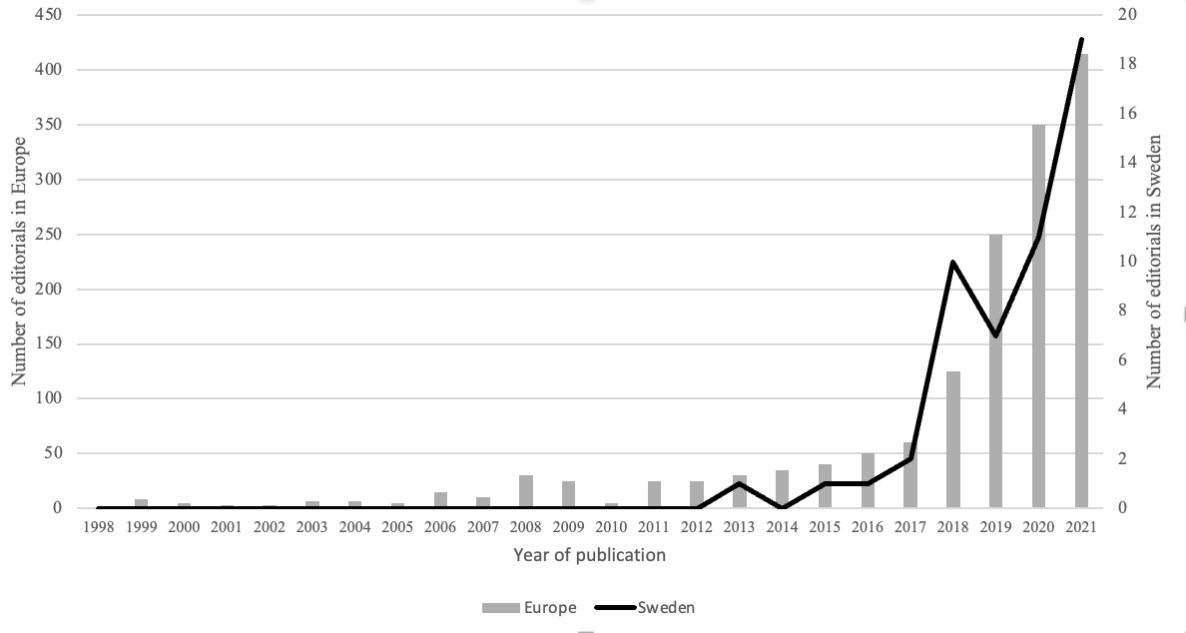
The number of published editorials in scientific journals related to artificial intelligence in health care in a Swedish and a European context (1998-2021).

To quantitatively assess the function strength, the number of editorials published in scientific journals was analyzed. As illustrated in Figure 4, the number of editorials related to AI innovations in a health care context has grown steadily (S9) over the last 4 years. The number of editorials in a Swedish context followed the same development as in a European context.

#### Entrepreneurial Experimentation (F5)

We assessed the entrepreneurial experimentation function (F5) by analyzing the number of medical disciplines involved in AI initiatives and the diversity of end user applications. On the supply side, the product mapping revealed that certain areas were more mature than others; for example, 182/343 (53%) products were in radiology, 31/343 (9%) products in cardiology, and 21/343 (6%) products in neurology. Moreover, the hospital departments (demand side) were actively engaged in a significant number of experiments related to AI (58 projects). The most prevalent categories were decision support (41%), diagnosis (21%), telemedicine (14%), monitoring (11%), and workflow improvements (8%). The remaining (4%) encompassed “smart” prosthetics, surgical robotics, and virtual reality glasses for rehabilitation. Hence, AI innovations were identified across medical disciplines and for various end user applications on the demand as well as the supply side (S10).

It is necessary to push, because you may not see the result so clearly now. But then we have to do motivational work, and you also need answers on ‘what’s in it for us?

Study participant H

Many interviewees expressed the belief that instead of being developed in-house, most upcoming AI products will be procured from external commercial entities. As a key factor in support of this perspective, several interviewees noted that the process of taking a product from research to regulatory approval was challenging. Despite generally being positive about commercial products, many respondents also highlighted the complicating factors associated with acquiring solutions from external actors, which included the importance of rigorous testing and validation to assess safety and efficacy.

If it works, it’s actually a breakthrough since we can neatly overcome the legal barriers that are currently causing a lot of trouble.

Study participant D

#### Market Formation (F6)

An examination of the external market reveals a growing number of available products and an increasing presence of commercial actors (S10). A total of 343 CE-approved products were identified, and the actors involved ranged from small startups to large corporations, totaling 284 different entities. Despite the theoretical potential of purchasing products that could bring immediate value, there appears to be a lack of a systematic process for these solutions to transition into clinical practice, as the number of implemented AI innovations in clinical practice was low (W7).

There are tons of apps out there and some people might wonder, why don’t we just buy them? But it’s not quite as simple as that.

Study participant B

In cases where no suitable product exists on the market, internal development may be pursued; however, the process of taking an algorithm from research to clinical practice was perceived as highly intricate. Despite a significant amount of AI research activities, the sentiment among respondents was that little, if any, of the research makes it to implementation, with a substantial percentage of pilot projects yielding no tangible outcomes. Concerns were expressed regarding the optimal integration of AI into established workflows and the need for alignment with existing clinical processes. A shared view emerged on the importance of rigorous testing and validation to ensure safety and efficacy prior to routine clinical implementation (W8).

We need to identify products and see which ones are valuable to us … we need support in the organization in terms of routines and testbeds to evaluate whether a certain product is useful or not ... otherwise, we do not want to buy this tool.

Study participant B

#### System-Wide Synergies (F7)

While the importance of laws and regulations, such as GDPR and MDR, in safeguarding privacy and security is acknowledged, some have argued that existing legislation can be overly restrictive (W9) and emphasize the importance of legislative alignment with technological advancements for the effective integration of AI innovations into clinical practice (W10).

In Sweden, we have the law of public procurement, which is not always the best method for this type of transformation. You specify your requirements and suppliers then have the opportunity to present offers. This is not particularly suitable for things that are somewhat fuzzy and difficult to define.

Study participant M

Founded in 2020, the National Center for AI Sweden was considered an asset to enhance collaboration across the ecosystem, fostering cluster-like dynamics (S11). The center is funded by the Swedish government through the Innovation Fund Vinnova, and its 120 partners represent private companies, the public sector, academia, and research institutes. The industry partners span sectors, such as automotive (China Euro Vehicle Technology AB, VOLVO AB, and Zenseact AB), computer science (Embedl AB, Recorded Future AB, and Talkamatic AB), and the life science industry (AstraZeneca AB and Essity AB). Fostering collaborations with diverse external actors provided a valuable exchange of knowledge; one participant also suggested expanding collaboration between regions to facilitate the sharing of ideas and best practices. The academic partners include Chalmers University of Technology and Gothenburg University. Participants felt that collaboration with academia was valuable in order to integrate AI elements into the education of future health care professionals.

I think it is very important to use our united forces.

Study participant G

### Summary of the Functional Assessment

Table 4 provides an overview of the functional assessments, encompassing both identified system weaknesses and strengths for the adoption of AI innovations in health care organizations. The number of weaknesses and strengths was used as an indication of function strength. If a function exhibited system strengths only, it was categorized as strong. A function showcasing more weaknesses compared to strengths was deemed weak. This demand-side analysis shows that guidance of search (F4) and entrepreneurial experimentation (F5) were strong functions, which may indicate functional interdependencies. Knowledge development and diffusion (F1) and legitimation (F2) showed intermediate strength. By contrast, resource mobilization (F3), market formation (F6), and system-wide synergies (F7) were assessed as weak.

It’s important to avoid narrowing down, and to instead obtain a comprehensive picture of the various factors. People sometimes tend to focus on one factor … but they go hand in hand.

Study participant G

**Table 4:** Assessment of functions based on identified strengths and weaknesses for the adoption of artificial intelligence innovations in a health care organization.

Identified strengths					Identified weaknesses
**Strong assessments**					
	**Function F4:** **guidance of search**				
		High expectations and strong belief in the strategic potential of AI^a^ within the hospital (S8).		N/A^b^	
		The number of editorials related to AI innovations in a health care context has grown steadily (S9).		N/A	
	**Function F5:** **entrepreneurial experimentation**				
		AI innovations were identified across medical disciplines and for various end user applications on both the demand and supply sides (S10).		N/A	
**Intermediate** **assessments**					
	**Function F1:** **knowledge development and diffusion**				
		Numerous research projects within the organization contributed to knowledge expansion (S1).		Lack of knowledge and expertise concerning the potential impact of AI at both the organization level and in everyday clinical practice (W1).	
		The number of publications in scientific journals has increased exponentially, which indicates continuous expansion of the general knowledge base (S2).		N/A	
	**Function F2:** **legitimation**				
		The Swedish government unveiled its national strategy for AI, emphasizing the need to harness the advantages and calling for active support to develop and adopt the strategy (S3).	The consensus-driven decision-making climate was viewed as potentially hindering transformative efforts (W2).		
		Positive attitude toward the usage and implementation of AI in health care (S4).	Uncertainties surrounding ethical considerations and patient data accessibility (W3).		
		The number of articles in Swedish daily newspapers increased exponentially, which indicates an increased social acceptance of AI innovations in a health care context (S5).	Weak transparency of data used for training the algorithms, resulting in uncertainties regarding the reliability of AI innovations (W4).		
**Weak** **assessments**					
	**Function F3:** **resource mobilization**				
		Obtaining funds for AI projects was straightforward, from the internal organization and from external sources (S6).	Concerns related to access to patient data, the adequacy of digital infrastructure, and interoperability of existing IT systems (W5).		
		N/A	Insufficient time to systematically explore potential AI applications (W6).		
	**Function F6:** **market formation**				
		A growing number of available products and an increasing presence of commercial actors (S10).	The number of implemented AI innovations in clinical practice was low (W7).		
		N/A	Importance of rigorous testing and validation to ensure safety and efficacy prior to routine clinical implementation (W8).		
	**Function F7:** **system-wide synergies**				
		The national center AI Sweden was considered an asset to enhance collaboration across the ecosystem, fostering cluster-like dynamics (S11).	Existing legislation may be overly restrictive (W9).		
		N/A	Legislative alignment with technological advancements for the effective integration of AI innovations into clinical practice (W10).		

^a^AI: artificial intelligence.

^b^N/A: not applicable.

### System-Blocking Mechanism and Functional Pattern

**Figure 5:**
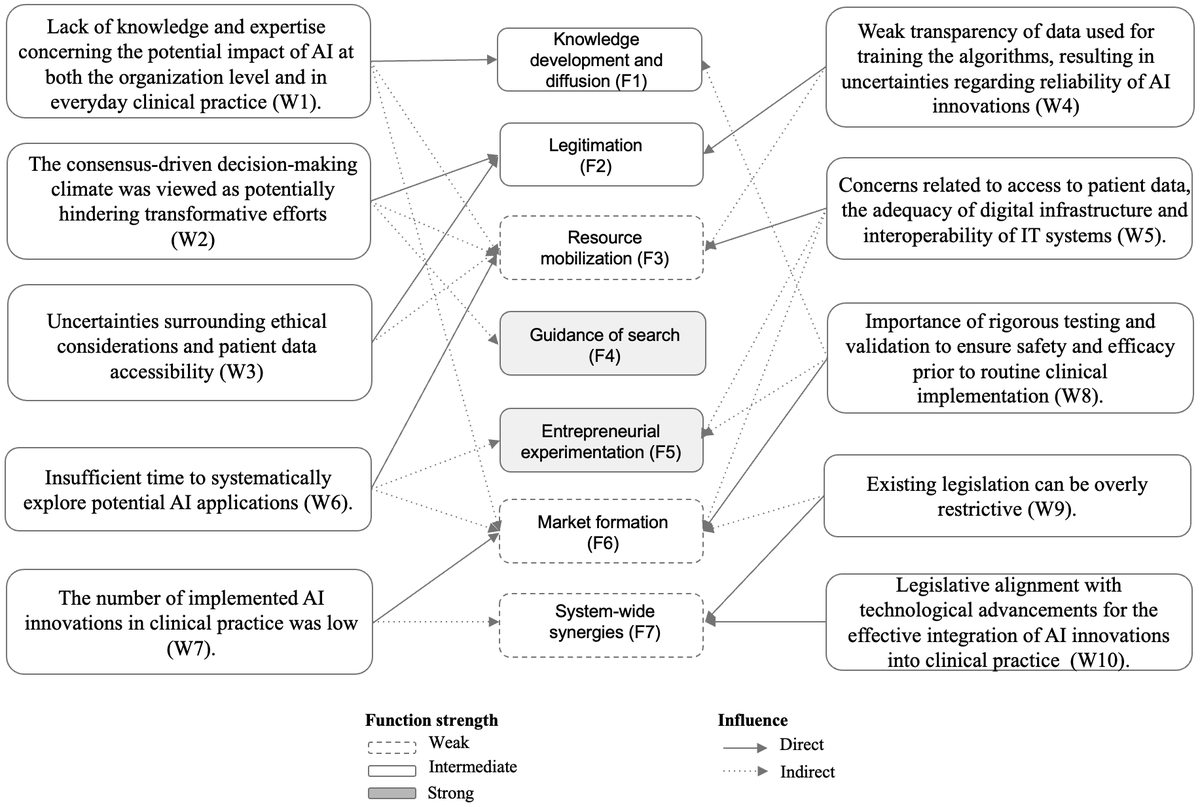
Function weaknesses or system blocking mechanisms for artificial intelligence and artificial intelligence–associated health care technology innovations. AI: artificial intelligence.

**Figure 6:**
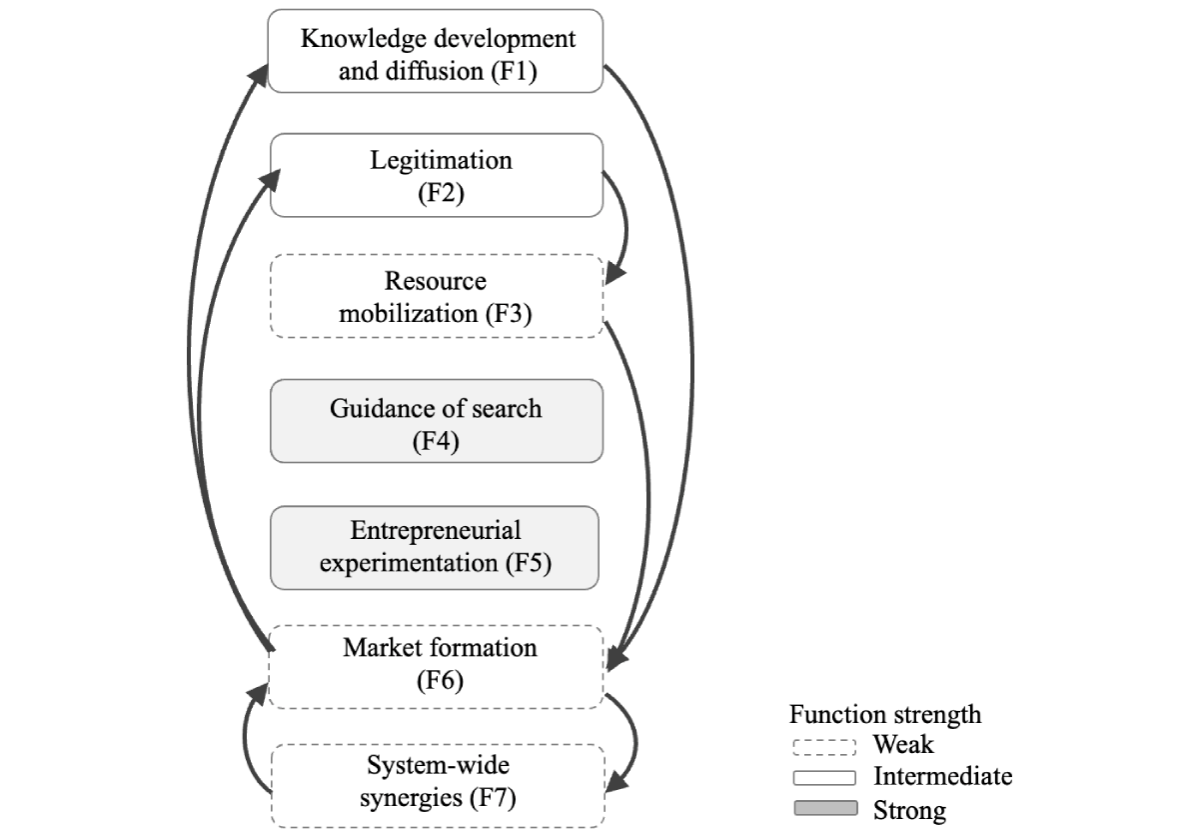
Illustration of the interactions and influences between the system functions.

Our analysis indicates that some weaknesses are more critical to address than others due to their direct and indirect effects on multiple functions, which block the system from developing further. The researchers conclude that strengthening knowledge development and diffusion (F1), legitimation (F2), and resource mobilization (F3) could trigger a cascade of positive activities, thereby significantly enhancing the overall performance of the innovation system. The identified patterns and interrelationships among these weaknesses are illustrated in Figures 5 and 6. Positive interactions between demand-side knowledge development (F1) and legitimation (F2) can generate virtuous cycles with resource mobilization (F3) and market formation (F6), thereby facilitating the adoption of new technologies. The uncertainties surrounding ethical considerations and patient data accessibility have a direct effect on legitimation but also discourage the allocation of resources, indicating that legitimation (F2) impacts resource mobilization (F3). Further, the lack of supportive mechanisms for ensuring the safety and efficacy of AI innovations has a direct influence on resource mobilization, and it also inhibits the implementation into clinical practice. The virtuous feedback loop from market formation (F6) to knowledge development and diffusion (F1) and legitimation (F2) is considered to be closely tied to the evidence-based culture in health care, where real-world testing and robust clinical evidence are prerequisites for the adoption of new innovations. Once safety and efficacy have been demonstrated, the knowledge and legitimacy of using these innovations increase, enabling further usage and integration into routine clinical practice.

## Discussion

### Principal Findings

This final part of the article discusses implications for policy and activities, together with some general conclusions. The main findings of this research are that if AI is to be broadly adopted by health care organizations, a number of factors need to be addressed. This study has especially highlighted that policy interventions and activities can target specific system-blocking mechanisms to enhance innovation system performance.

### Implications for Policy and Strategic Management Decisions

The findings of this study indicate that adopting AI technology innovations in health care organizations can be accelerated through targeted strategies and supportive policy frameworks that facilitate clinical validation and create compelling use cases. To facilitate adoption, policymakers should address legislative uncertainties regarding ethics and patient data access, including digital infrastructure, interoperability across IT systems, and improvements in data quality and transparency. Moreover, creating dedicated testing environments to evaluate safety and efficacy is essential for building trust in the technologies and enabling clinical integration. A feedback loop from market formation to knowledge development and legitimation is closely tied to the evidence-based culture in health care, where real-world testing and robust clinical evidence are prerequisites for the adoption of new practices. Robust clinical evidence not only informs stakeholders about AI’s potential but also strengthens legitimacy among clinicians, regulators, and payers. To reinforce knowledge development and diffusion, a broader understanding of AI needs to permeate the demand-side organization, coupled with sustained collaboration efforts. The role of driven individuals throughout the operation, acting as pioneers in their respective professions, was emphasized as vital in this endeavor.

Our findings should also be considered in light of the broader challenges health care organizations face when adopting new technologies. Drawing on quality improvement theory in health care, adoption decisions require managing the tensions and the often competing priorities of patients (safety and access), providers (clinical autonomy), and payers (cost control) [38-40]. In the context of AI adoption, transparency and performance monitoring are essential to align stakeholder interests and ensure safe, legitimate use. This tension is particularly relevant for AI, where the opacity of underlying models complicates both trust and accountability. Within the TIS framework, such challenges are closely related to the legitimation and knowledge functions: transparency tools and robust performance monitoring are not only critical for safeguarding patients and ensuring responsible resource allocation but also for strengthening societal acceptance and supporting the diffusion of credible knowledge about how these technologies perform in practice. By reinforcing these system functions, transparency and monitoring mechanisms become enablers of both safe adoption and long-term integration of AI in health care.

### Theoretical Contribution

This study has demonstrated the utility of the TIS in analyzing the dynamics within an innovation system specific to the health care sector, with a primary focus on a particular health care organization. Consistent with previous studies [5,15,16,25], our findings suggest that positive interactions between system functions can generate reinforcing dynamics within the TIS, triggering virtuous cycles that facilitate the adoption of new technologies. In the health care context, for example, clinical validation of a new technology increases knowledge development and enhances its legitimacy, which in turn attracts additional resources. As resource availability increases, so does the adoption and use of technology. Consistent with previous studies of health care innovation systems [5,25], our analysis indicates the interdependence of guidance of search (F4) and entrepreneurial experimentation (F5), which provide the initial conditions for knowledge development and diffusion (F1) and legitimation (F2). Further, our analysis indicates the interdependence of resource mobilization (F3) and market formation (F6), which, when strengthened, provides the initial conditions for system-wide synergies (F7). Given that this study is a single-case analysis, further research is essential to assess and expand upon the functional dynamics within health care contexts.

### Limitations

A limitation of this study concerns its generalizability, given its reliance on a case study with a restricted dataset. Caution should be exercised when extrapolating the findings and drawing conclusions. Despite this, several aspects might be relevant to other contexts, particularly within health care organizations. The study’s interviews were somewhat limited in number, and the participants, being individuals who are interested and knowledgeable about AI, may not offer a fully representative perspective of the entire organization. This selection was intentional, seeking to extract pertinent information efficiently from a confined interview pool. However, a broader range of interview participants, including IT professionals and individuals with legal expertise, and patients, could also have added value from the ethical dimensions of AI in health care.

## Abbreviations

AI: artificial intelligence
CE: Conformité Européenne
CHAIR: Chalmers Artificial Intelligence Research Centre
EU: European Union
GDPR: General Data Protection Regulation
MDR: medical device regulation
OECD: Organisation for Economic Co-operation and Development
RISE: Research Institutes of Sweden
SFS: Svensk författningssamling
TIS: Technological Innovation Systems
WHO: World Health Organization
